# Supplementary material for: Are carbon emissions trading and green financial instruments synergistic? -Comprehensive quantitative research based on content analysis
Source: PLoS One. 2024 Mar 7;19(3):e0298601. doi: 10.1371/journal.pone.0298601 (PMC10919601; doi:10.1371/journal.pone.0298601)
Supplement: S1 Data — (ZIP) [file pone.0298601.s001.zip › Data/地级市Z_F工作报告.zip!/║■─╧/╒┼╝╥╜τ╩╨/└┤╫╘╣1⁄2╓┌║┼í╛┬φ┐╦╩2╛▌═°í┐_2018.docx]

各位代表：

　　现在，我代表市人民政府向大会报告工作，请予审议，并请各位政协委员及其他列席人员提出意见。

　　一、2017年工作回顾

　　2017年，我们在省委、省政府和市委的坚强领导下，全面落实新发展理念，深入实施“对标提质、旅游强市”战略，按照“11567”总体思路，突出打好脱贫攻坚、转型升级和环境治理“三大战役”，全力稳增长、促改革、调结构、惠民生、防风险，各项工作取得新的成效。

　　（一）经济运行稳中有进。坚持以推进供给侧结构性改革为主线，致力供需两端发力，认真开展“项目建设年”“产业发展年”“旅游服务年”活动，主要经济指标平稳较快增长。全市实现地区生产总值550亿元（预计数，下同），增长8.5%；完成固定资产投资350亿元，增长14.8%；完成一般公共预算收入55.49亿元，增长12.14%；城乡居民人均可支配收入分别达22900元、8700元，分别增长8.9%、11.5%，五项指标增速均高于全省平均水平，其中地区生产总值、固定资产投资、一般公共预算收入增速位居全省前列。消费市场保持平稳，实现社会消费品零售总额219亿元，增长10.6%。

　　（二）全域旅游基地建设成效明显。加快国家全域旅游示范区建设，推进旅游业转型升级、提质增效，全省旅游龙头作用进一步凸显。全年接待游客7250万人次、过夜游客3450万人次，分别增长18%和21%，实现旅游总收入620亿元，增长21%。“三星拱月、月照三星”全域旅游发展布局不断优化，核心景区提质升级步伐加快，东、西、南线发展统筹推进，大庸古城、西线旅游、冰雪世界、桃花溪谷、七星山开发等项目扎实推进，万豪路上汽车酒店、万福温泉改造等项目建成营业，禾田居山谷、五号山谷、石堰坪等乡村旅游快速发展，全域旅游新业态不断涌现，旅游产品更加丰富。旅游营销不断创新，在哈萨克斯坦等“一带一路”沿线国家建立了丝路驿站，赴东南亚、欧美等多个国家和地区开展了友好交流，第二届中国张家界民俗文化月、东北亚地区地方政府联合会议、世界跑酷大赛、冬游张家界、国际旅游诗歌节等活动成功举行，张家界知名度和影响力持续扩大。旅游服务水平不断提升，游客中心、旅游厕所等旅游设施提质升级，“智慧旅游”体系加快建设，“全域通”全面上线，“平安满意在张家界”专项活动深入开展，旅游行业协会自律逐步加强，旅游满意度和美誉度持续增强。

　　（三）产业转型升级步伐不断加快。突出旅游带动，推进产业融合，产业结构进一步优化，三次产业结构比为10.7：20.6：68.7。大力发展新型工业，加快建设“三区一园”，完成园区基础设施建设投资6亿元、新增入园企业18家，新开工工业项目24个，竣工投产16个。完成工业技改投资43亿元，新增入规企业25家、高新企业15家、过亿元企业10家，规模工业增加值增长6.3%。大力发展现代农业，农业“523”行动计划圆满完成，大鲵发展到212万尾、商品蔬菜基地达到31.5万亩。培育新型农业经营主体，新增土地流转面积4万亩、农民专业合作社221家，农业增加值增长3.5%。大力发展现代服务业，义乌国际商贸城落户建设，奇峰、黄金塔等城区农贸市场项目稳步推进，7个农村集贸市场建成使用；交通银行成功进驻，金融机构存贷款余额增幅居全省第一；市电子商务运营服务中心建设全面启动，会展、物流和汽车服务业快速发展，新增电商企业96家、网络经营主体603户，会展销售达到6.11亿元。

　　（四）城乡面貌持续改善。以“六城同创”为抓手，以“三带三区”“三化三改”为重点，以国际化理念推进规划建设管理，城乡面貌不断改观。强化城市规划管控，《张家界市城乡规划技术规定》修订完成，枫香岗、荷花等六个组团及片区控规编制工作全面开展，城市设计编制工作加快推进。完善城市大交通，张桑高速建成通车，邢大公路基本建成，黔张常铁路、高铁西站、武陵山大道、杨家界大道、子午西路延伸段建设步伐加快，张吉怀高铁顺利开工，旅游观光磁浮专线前期工作扎实推进。推动城市品质不断提升，沙堤大道路基完成，机场北路、融山东路、澧水大桥南延高架桥、西溪坪东互通立交改造、天马路建成通车，杨家溪体育公园、白马泉高架桥滨河景观工程、高云公园建成开放，4个智能化公共停车场基本建成，共享汽车、共享单车投入使用。加强城市管理，市容环境、交通秩序整治初见成效，“垃圾出城”行动成效明显，“征地拆迁清零”活动深入开展，完成棚户区改造11161户187万平方米，城市精细化管理水平不断提升。统筹推进县城和小城镇建设，慈利老城区改造及蒋家坪新区建设、桑植老观潭新区建设步伐加快，天子山、三官寺、洪家关、教字垭特色旅游风情镇基本建成。

　　（五）改革开放取得新进展。深入推进“放管服”改革，承接、取消、调整、下放行政权力事项155项，清理公共服务事项230项、行政审批中介服务事项45项。持续深化商事制度改革，新增市场主体1.45万户，完成“个转企”491家、“小升规”65家。不动产登记、农村土地承包经营权确权、医疗卫生、投资体制、社会保险等改革稳步推进，武陵源门票价格分成机制取得新突破，旅游统计改革卓有成效。扎实开展“双创”工作，2家众创空间通过省级认定，慈利县、桑植县获批国家级电子商务进农村综合示范县，“硒有慈利”成为全省首个县域农产品公用品牌。大力发展开放型经济，张家界航空口岸获批“境外游客购物离境退税试点”，丰泽公共保税仓开工建设，“四个进境指定口岸”建设顺利推进，国家级生态原产地产品保护示范市、桑植县出口农产品质量安全示范县成功创建。招商引资取得实效，利用外资1.18亿美元，增长15.9%；实现内联引资59.06亿元，增长18%。实现外贸进出口8800万美元，增长10%。

　　（六）民生福祉不断提升。民生支出占一般公共预算支出比重达77.61%，完成了30件43个省市重点民生实事项目。全力推进脱贫攻坚，扎硬寨、打硬仗，完成省级考核督查交办88个突出问题集中整改，顺利通过国家脱贫攻坚巡查组验收；完成易地扶贫搬迁8936人、农村危房改造8030户、贫困村电网改造47个，解决5.64万贫困人口饮水问题，“阳光三保障工程”扎实推进，90个村退出贫困村序列，4.9万贫困人口实现脱贫。着力强化社会保障，新增城镇就业1万人、农村劳动力转移就业1.36万人，养老、医疗、生育、工伤和失业保险覆盖面进一步扩大。加快发展社会事业，张家界市一中新校区、张家界民族小学竣工使用，完成22所学校“全面改薄”任务，建成农村义务教育标准化教学点64个。市人民医院新院等项目加快推进，建成9所合格乡镇卫生院、4个社区卫生服务中心和483个村卫生室，计划生育工作水平不断提升。大力推进公共文化服务体系建设，送戏下乡等文化惠民行动扎实有效，“一宫四中心”项目加快实施，新增规模以上文化企业13家、村级综合文化服务中心示范点30个。农村综合服务平台加快建设。着力加强生态建设，划定并严守生态保护红线，全面落实河长制，深入推进规范农民建房、集镇环境整治、旅游干线环境整治、环境整治“6+1”四个专项行动，大力开展环境治理战役，中央环保督察组反馈问题基本整改到位、交办信访件全部办结，核心景区环境整治取得明显成效，城市空气质量优良天数达到321天，成功创建国家森林城市、省级园林城市。深入推进“平安张家界”建设，扎实开展食品安全示范城市创建，严格落实安全生产“一岗双责”和隐患排查治理“一单四制”，安全生产工作被评为全省优秀单位；持续开展信访积案攻坚化解活动，成功化解一批信访积案和历史遗留问题；纵深推进“围猎”专项行动和交通违法行为专项整治“百日会战”，严厉打击各类违法犯罪，社会大局保持和谐稳定，第三次捧得全国综治最高奖“长安杯”。

　　（七）政府自身建设全面加强。推进“两学一做”学习教育常态化制度化，开展“凝心聚力、开放奋起”解放思想大讨论活动，施政能力和服务水平进一步提升。加快推进“互联网+政务服务”，出台市级政府投资、市政项目建设、政府法律顾问等管理办法及制度，行政效能进一步提升。严格落实党风廉政建设各项规定，驰而不息纠“四风”，严肃查处违反中央八项规定案件，通报曝光“四风”问题129人次。自觉接受人大及其常委会法律监督、工作监督和政协民主监督，省、市165件人大代表建议和127件政协提案全部办结。加强行政监察和审计监督，严格绩效考核，政府执行力和公信力进一步提升。

　 　　一年来，国防和后备力量建设得到加强，编制、统计、民族、宗教、侨务、对台、人防、烟草、档案、史志、气象、水文、残联、移民、应急管理等工作实现新进步，国税、地税、电力、通信、金融等驻张单位为全市经济社会发展作出新贡献，各民主党派、工商联、无党派人士及工会、共青团、妇联、科协、侨联等人民团体的工作取得新成绩。

　　各位代表，过去一年的成绩来之不易，值得倍加珍惜。这些成绩的取得，是省委、省政府和市委正确领导的结果，是市人大、市政协有效监督、鼎力支持的结果，更凝聚了全市人民的智慧和汗水。在此，我代表市人民政府，向全市各族人民，向人大代表、政协委员，向各民主党派、工商联、无党派人士和人民团体，向驻张部队、武警官兵和政法干警，向所有关心支持张家界发展的各界朋友，表示衷心的感谢并致以崇高的敬意！

　　在总结成绩的同时，我们也清醒地看到，我市经济社会发展仍面临不少困难和问题：工业经济短板明显，三次产业结构有待优化，产业聚合度不高，经济发展的质量和效益有待进一步提高；出市大通道以及域内交通网络有待完善，城乡基础设施建设有待加强；景区管理、旅游市场监管、城市管理有不少盲点和薄弱环节，与国内外知名旅游胜地的要求还有较大差距；脱贫攻坚的任务还很重，民生改善力度需进一步加大；创新能力不足、人才支撑亟待加强；少数干部创新意识、担当精神不强，行政效能有待进一步提升，等等。对此，我们将直面问题、不遮不掩，采取有力措施努力加以解决。

　　二、用党的十九大精神统揽政府工作

　　党的十九大是在全面建成小康社会决胜阶段、中国特色社会主义进入新时代的关键时期召开的一次具有里程碑意义的重要会议，习近平总书记所作的党的十九大报告举旗定向、高屋建瓴、博大精深、求真务实，凝聚全党智慧，反映时代要求和人民愿望，是党和国家各项事业的基本遵循。学习宣传贯彻党的十九大精神是当前和今后一个时期的首要政治任务，做好政府工作必须坚持以党的十九大精神为统揽，切实把党的十九大精神贯彻落实到政府工作各方面和经济社会发展全过程。

　　——必须旗帜鲜明坚持党的领导。中国特色社会主义最本质的特征是中国共产党领导。做好我市政府工作，要不断强化践行“四个意识”的思想自觉和行动自觉，始终在思想上政治上行动上与习近平同志为核心的党中央保持高度一致，始终与党委同心同向、同频共振，坚决贯彻省委“创新引领、开放崛起”战略，坚决落实市委“对标提质、旅游强市”战略和“11567”总体思路，扎实推进“城市扩容提质、产业转型升级、交通综合提升、城乡环境同治、脱贫决胜攻坚、民生补短强基”三年行动计划，以务实有力的举措确保市委的决策部署得到贯彻落实。

　　——必须以习近平新时代中国特色社会主义思想为行动指南。习近平新时代中国特色社会主义思想是我们党理论创新的重大成果，是当代中国的马克思主义。做好我市政府工作，必须以习近平新时代中国特色社会主义思想武装头脑，指导实践，推动工作，紧扣经济社会发展新目标，把握社会主要矛盾新变化，正确处理当前与长远、发展与保护、速度与质量、经济发展与民生改善、旅游发展与跨界融合的关系，努力开创全市经济社会发展新局面。

　　——必须始终坚持以人民为中心的发展思想。增进民生福祉是发展的根本目的。做好我市政府工作，必须始终把人民利益摆在至高无上的地位，着力解决人民日益增长的美好生活需要和不平衡不充分的发展之间的矛盾，按照兜底线、织密网、建机制的要求，加快建立多层次社会保障体系，统筹发展社会事业，确保在幼有所育、学有所教、劳有所得、病有所医、老有所养、住有所居、弱有所扶上持续取得新进展，让人民群众的获得感、幸福感、安全感更加充实、更有保障、更可持续。

　　——必须把改革创新贯穿始终。改革是解决前进道路上困难和问题的关键，创新是引领发展的第一动力。做好我市政府工作，要牢牢把握新常态大逻辑，全面贯彻新发展理念，努力破除影响发展的思维定势、行为惯性、路径依赖，勇于变革、勇于创新，主动融入国家“一带一路”建设、长江经济带战略和省“创新引领、开放崛起”战略中去，深化供给侧结构性改革、要素市场化配置改革，建立技术创新体系，推动经济发展质量变革、效率变革、动力变革，不断增强经济社会发展的创新力和竞争力。

　　——必须把依法行政作为政府工作准则。依法行政是贯彻依法治国方略、提高行政管理水平的基本要求。做好我市政府工作，必须牢固树立法治意识，把政府工作全面纳入法治化轨道，忠实履行宪法和法律赋予的职责，不断提高依法行政的能力和水平，运用法治思维和法治方式深化改革、推动发展、改善民生、化解矛盾、维护稳定。

　　——必须把防风险、守底线作为政府工作的重要职责。坚持底线思维，增强驾驭风险本领，才能牢牢把握工作主动权。防范金融风险、严控政府债务、守住绿水青山、维护安全稳定、保持廉政本色，这是做好我市政府工作必须坚守的底线，也是义不容辞的责任。必须统筹好谋发展与防风险、保稳定的关系，确保全市安全发展、绿色发展、可持续发展，确保全市社会大局和谐稳定。

　　三、2018年工作安排

　　2018年，是贯彻落实党的十九大精神的开局之年，是打赢脱贫攻坚战、决胜全面建成小康社会的关键之年，是改革开放40周年，也是我市建市30周年，做好今年工作意义重大。纵观发展形势，国际环境正在发生深刻复杂变化，全球经济将延续复苏态势，但不确定、不稳定因素依然存在。国内经济已由高速增长阶段转向高质量发展阶段，正处于转变发展方式、优化经济结构、转换增长动力的攻关期，改革发展仍然面临不少困难和挑战。我市作为后发地区，基础差、底子薄的基本市情没有根本改变，依然面临发展不足与发展不优的双重矛盾、资源与环境的双重约束、加快发展与加快转型的双重压力。我们必须保持清醒认识，切实增强忧患意识。同时，我们更要看到发展新机遇。中国特色社会主义进入新时代，国家实施区域协调发展战略、乡村振兴战略，建立市场化、多元化生态补偿机制，支持革命老区、民族地区、贫困地区加快发展，支持实体经济发展，支持棚户区改造。省里继续支持大湘西、武陵山片区区域发展与扶贫攻坚，加快推动旅游资源大省向旅游强省跨越，建设张吉怀生态文化旅游经济带，支持张家界在“锦绣潇湘”全域旅游基地建设中发挥龙头作用。我市高速高铁加快建设、航空口岸加快发展、全域旅游扎实推进，正处于提质升级、加快发展的黄金期，经济社会必将迎来新一轮大发展、大跨越。全市上下必须把握大势，抢抓机遇，顺势而为，奋力夺取今年各项工作新胜利，不断开创全市经济社会发展新局面。

　　今年政府工作的总体要求是：全面贯彻党的十九大精神，以习近平新时代中国特色社会主义思想为指导，认真落实新发展理念和中央、省委、市委经济工作会议精神，坚持稳中求进工作总基调，紧扣社会主要矛盾变化，按照高质量发展的要求，统筹推进“五位一体”总体布局，协调推进“四个全面”战略布局，以供给侧结构性改革为主线，以产业发展为根基，以项目建设为抓手，以创新开放为动力，着力抓重点、补短板、强弱项，深入实施“对标提质、旅游强市”战略和“11567”总体思路，扎实推进“三年行动计划”，统筹做好稳增长、促改革、调结构、惠民生、防风险各项工作，在打好防范化解重大风险、精准脱贫、污染防治攻坚战方面取得扎实进展，推动经济社会持续健康发展，以优异的成绩迎接建市30周年。

　　今年全市经济社会发展的主要预期目标是：地区生产总值增长 8.5%以上，规模工业增加值增长7%以上，固定资产投资增长15%以上，旅游总收入增长20%以上，社会消费品零售总额增长11%以上，一般公共预算收入增长10%以上，城镇居民、农村居民人均可支配收入分别增长10%和11%。社会治安综合治理、计划生育、安全生产、节能减排等完成省定任务。

　　围绕上述要求和目标任务，重点抓好以下九个方面工作：

　　（一）做精做美全域旅游，增强旅游综合竞争力。对标国际国内一流旅游目的地，充分发挥绝版旅游资源优势，以国际化视野谋划和推动旅游业的转型升级和品质提升，着力推进旅游业标准化、精细化、信息化和国际化，努力在旅游产品供给、国际精品旅游城市建设、旅游跨界融合、旅游管理体制机制创新、旅游国际化、旅游惠民富民、旅游辐射带动七个方面当龙头作示范，在全国全省率先建成国家首批全域旅游示范区。要在完善全域旅游格局上迈出新步伐。按照“三星拱月、月照三星”发展思路、“双核三极多点”产业布局，突出武陵源核心地位，全面实施核心景区旅游设施提质，打造环景区乡村旅游带，东线重点整顿大峡谷周边环境、加快衍生产品开发，西线重点加快茅岩河、九天洞景区以及红色旅游项目建设，南线重点推进环天门山景区开发。要在丰富旅游产品供给上迈出新步伐。建成桃花溪谷、冰雪世界、九天峰恋、红军体验园等项目，加快建设大庸古城、武陵古镇、七星山旅游开发、张家界俄罗斯国家马戏城、张家界千古情等项目。积极引进国际知名品牌酒店，鼓励发展特色精品民宿。围绕打造千里旅游观光走廊，加快乡村旅游产品建设。推进“旅游+”深度融合，培育发展文化旅游、体育旅游、健康旅游等新业态新产品。要在推进旅游国际化上迈出新步伐。以主要客源地和交通枢纽城市为重点，巩固东亚、港澳台等传统境外市场，开发东南亚、欧美等新兴境外市场。加强与国际旅游组织、国际友好城市、国家旅游局驻外机构的联系，深化国际旅游交流，加强与丝绸之路国际总商会的合作，拓展丝路驿站，办好东北亚地区地方政府联合峰会、国际旅游诗歌节。完善对外营销体系，建立多语种旅游宣传网站和海外推广机构，支持旅游企业参加国际旅游活动，积极争取更多国际性会议和体育赛事落户张家界，加快推进友城和对外友好交往工作，扩大国际影响力。要在提升旅游服务管理水平上迈出新步伐。加强旅游研究，规范旅游管理，推进旅游管理服务标准化。加快“智慧旅游”建设，完善“全域通”信息系统，建好旅游大数据平台，加快旅游信息化进程。坚持不懈推进旅游“厕所革命”，开工建设旅游运输综合服务管理中心，加快建设游客服务中心。深化“平安满意在张家界”专项活动，推进发票门票“一票制”、旅游佣金“公对公”改革，完善“1+3+N”旅游综合管理和综合执法体系，加大旅游市场整治力度，不断优化旅游市场环境。

　　（二）全力推进产业转型升级，加快构建现代产业体系。认真贯彻省委“产业项目建设年”的部署，扎实推进“产业发展年”活动，坚持不懈抓重点、补短板、强弱项，着力推动工业、农业、服务业的提质、扩能、增效，促进经济结构优化。加快推进新型工业化。以园区为平台，致力打造农产品加工、生物医药、旅游商品加工、信息与高端装备制造“四大工业支柱产业”，狠抓园区载体，做强园区经济，支持张家界经开区创建国家级高新区。完善产业布局，优化功能分区，推动“三区一园”错位发展。突出项目支撑，推动康美大德、三诺智能、富士莱手机、湘丰茶叶产业园等项目落地投产，做大做强久瑞生物、康尔佳药业。进一步完善园区管理体制，加快园区基础设施建设，优化园区管理服务，营造优良发展环境，全年园区工业增加值占全部规模工业增加值比重达到45%以上。加快发展现代农业。以规模化、特色化、品牌化发展为核心，坚持以工促农、以商活农、以旅带农、科技强农、规模兴农，致力发展大鲵、蔬菜、茶叶和水果“四大农业特色产业”，优化产业结构，新增大鲵60万尾，确保商品蔬菜基地、茶园和水果种植面积分别达到32万亩、12.5万亩和56万亩。加快构建现代农业生产体系、经营体系和服务体系，组建市农业投资公司，加大对农业投入，吸引工商资本进入农业产业，发展适度规模经营，培育新型农业经营主体，培养1000名新型职业农民，农民专业合作社发展到1600家。加强“茅岩莓”“桑植白茶”“硒有慈利”品牌培育。支持永定农业科技园创建国家级园区。提高现代服务业发展水平。致力构建电子商务、现代物流、现代金融和会议会展“四大服务产业”，做大做强现代服务业。大力发展电子商务，实施农村电商“个十百千万”工程，支持慈利、桑植国家电子商务农村综合示范县创建工作。着力发展现代物流业，完善仓储设施，发展冷链物流，建设张家界物流园。加快发展现代金融业，积极引进金融机构，大力创新金融产品。加快发展会议会展经济，改造提升商贸零售业，开工建设农特产品交易中心、宁邦国际广场，加快建设吃遍中国、义乌国际商贸城，建成武陵山珍馆，改建10家农贸市场，更好满足城乡居民消费需求。大力发展通用航空业和低空旅游，规划建设临空产业园，争创国家通用航空示范区。

　　大力发展实体经济。推动互联网、大数据、人工智能和实体经济深度融合，在中高端消费、绿色低碳、现代供应链、共享经济、会展经济等领域培育新增长点，形成新动能。对接融入“中国制造2025”、制造强省“1274”行动，打造农产品加工、生物医药、旅游商品加工等产业链，提升价值链，部署创新链，完善资金链。支持非公有制经济发展，营造平等竞争、健康发展的市场环境。加强产学研结合，设立产业发展引导基金、孵化基金，助推各类企业梯次发展，全面完成“个转企、小升规”三年行动计划，年内高新技术企业达到20家以上。深入开展“质量兴市”活动，强化企业家“以质取胜”战略意识，引导民营企业建立现代企业制度，支持企业主导或参与制定各类标准，推进全产业链标准化生产，弘扬工匠精神，增强企业核心竞争力。实施品牌战略，挖掘传承老字号品牌，培育区域公用品牌，新增注册商标500件以上。树立正向激励导向，营造企业家健康成长环境，培育企业家队伍，弘扬优秀企业家精神，更好发挥企业家作用。

　　（三）做实做细扶贫攻坚，坚决打赢脱贫攻坚战。坚持大扶贫格局，继续以桑植县为主战场，深入实施脱贫攻坚“1332”行动计划，紧扣“三率一度”，保证现行标准下的脱贫质量，既不降低标准，也不吊高胃口，做到脱真贫、真脱贫。改善基础设施。开工建设竹黄、三人、瑞中、定大、澧洪、G353慈利至市城区等干线公路，加快西线旅游交通建设，提质改造400公里农村公路，实施自然村通水泥（沥青）路工程，完成107个村农网改造任务，确保农村自来水普及率达到78%以上。推进产业扶贫。注重扶贫与扶志、扶智相结合，激发贫困人口内生动力。鼓励贫困乡村利用生态优势和传统文化优势，建设各具特色的美丽乡村，发展乡村旅游，打造旅游精准扶贫的“张家界样本”。进一步抓好金融扶贫、光伏扶贫、生态补偿扶贫和劳务输出扶贫。支持贫困乡村因地制宜发展特色产业，努力实现“一乡一业、一村一品”目标。推广永定区“一亩园经济”做法，大力发展庭院经济。加大保障力度。完善责任落实机制、督查巡查机制、考核问责机制，压实工作责任。抓好涉农资金整合，多渠道筹措资金，加快项目建设进度，加强扶贫资金管理，提高资金使用效率。继续实施“阳光三保障工程”，不让一个学生因贫失学，有效防止“因病致贫、因病返贫”现象发生，完成17380人易地扶贫搬迁、5000户危房改造和400户“阳光院”建设任务。全面完成年度脱贫攻坚任务，确保180个贫困村退出、7.6万贫困人口脱贫，永定区、慈利县整体脱贫摘帽。

　　（四）坚持项目带动，持续扩大有效投资。以项目承载投资，以投资推动发展，继续开展“项目建设年”活动，突出抓好十大产业项目、十大基础设施项目、十大社会民生项目，掀起项目建设新高潮，努力在全省“5个100”工程中实现新作为。加快重点项目建设。全力抓好黔张常铁路、张吉怀高铁和安慈高速建设，力争桑龙高速开工，扎实推进张石铁路、张新高速、宜张高速等前期项目，积极推进旅游观光磁浮专线项目。统筹推进产业、民生、社会发展项目建设。切实加强项目管理，落实项目建设“五个一”、“一单三制”、重点项目联席会议等制度，推行工程总承包模式，提高项目建设水平。扩大招商引资实效。坚持大招商、招大商，积极开展对接500强、对接新丝路、对接北上广、对接自贸区、对接大湘商活动，精心策划、包装一批重大项目，力争引进一批战略投资者，支持发展总部经济。依托“沪洽周”“湘品出湘”等招商平台，强化专题招商和产业链招商。开展“引老乡、回故乡、建家乡”行动，吸引优秀人才返乡建功立业。发挥异地商会桥梁作用，大力开展以商招商。落实招商项目责任制，完善“统分结合、归口管理”机制，形成招商引资合力。推行“一站式”服务，确保招商项目落地。着力强化要素保障。加强税源涵养，调整财税结构，提升财政收入质量。突破传统融资方式，运用现代金融手段融资，用好贫困县IPO绿色通道政策，吸引外地企业来张落地上市，培育本地优势企业上市。建立“政银保担投”联动机制，组建担保集团，破解融资难题。放宽民营资本市场准入，扩大融资规模。加强土地开发整理，依法推进征地拆迁，拓展用地空间，盘活土地存量，加强土地集约节约使用，最大限度发挥土地使用效益。加快引进培养高层次创新型人才，提供强有力的人才和智力支撑。严厉打击强行参工参运、恶意阻工等行为，营造项目建设良好环境。

　　（五）致力国际精品旅游城市建设，推进城乡融合发展。坚持用国际化理念规划城市，用国际化标准建设城市，用国际化规则管理城市，着力建设进出方便、安全快捷的“畅达城市”，宜游宜养、舒适舒心的“休闲城市”，山清水秀、天朗地净的“生态城市”，全域覆盖、智能高效的“智慧城市”，诚信友善、和美品质的“文明城市”，加快推进国际精品旅游城市建设。完善城乡规划体系。推进“多规合一”工作及平台建设，深化空间规划研究，建立三维仿真辅助决策系统。完成城市总体规划修改、武陵源风景名胜区总体规划修编等规划编制，基本实现村庄规划“全覆盖”。统筹推进城市“双修”、农村“双改”，加强建筑体量、风貌、色彩、高度、容积率等规划要素管控。进一步强化项目批后监管，确保规划落实到位。推进控违拆违工作，实现违法建设“零增长”。全面提升城市品质。按照“改造一片、建成一片、启动一片”思路，改造官黎坪、南庄坪、永定组团，加快建设沙堤、且住岗组团，启动荷花、枫香岗组团和茅溪片区建设，加快城市提质扩容步伐。围绕“城市环线、景区环线、滨水环线”，抓好10条主次干道、“八桥两隧”建设，建成武陵山大道、杨家界大道、索溪大道和子午西路延伸段，完善城市路网体系。围绕“微建设、微管理、微服务”，建设五子坡等6个公园、东门桥等3条地下通道、10个公共停车场，实施8条道路雨污分流管网改造，新建改建30个公共厕所，推进城市慢行系统和农贸市场体系建设，建成一批小游园、垃圾收运设施；改造棚户区13085户256万平方米，建成一批限价商品房；加快“智慧张家界”建设，推进“六城同创”，巩固国家森林城市创建成果，实施城市绿化、亮化、美化工程，开工建设静脉产业园，推进“公交都市”创建，加强共享汽车、共享单车应用管理，加大小区物业综合管理力度，提升城市宜居宜业宜游水平，让城市更加贴心、更有温度。推进县城和小城镇建设。支持慈利撤县设市。以慈利县城新区、桑植老观潭新区为重点，推进县城扩容提质。大力提升重点镇发展水平，引导发展阳和体育小镇等特色镇，形成助推县域经济发展的重要增长极。

　　大力实施乡村振兴战略。坚持农业农村优先发展，全面贯彻落实“产业兴旺、生态宜居、乡风文明、治理有效、生活富裕”的总要求，加快推进农业农村现代化。巩固和完善农村基本经营制度，深化农村土地制度改革，严格落实农村土地承包经营权延长30年的政策，积极推进农村土地 “三权分置”。深化农村集体产权制度改革，盘活农村集体资产，增强集体经济活力。促进生产要素城乡双向流动，推动“资源变股权、资金变股金、农民变股民”改革，建设田园综合体、农村产业融合发展示范园，带动农民收入稳定增长。推动农村基础设施提档升级，逐步缩小城乡基础设施差距。全面完成村级综合服务平台建设，逐步建立全民覆盖、普惠共享、城乡一体的基本公共服务体系，让符合条件的农业转移人口在城镇定居落户。创新乡村治理体系，建立健全党委领导、政府负责、社会协同、公众参与、法治保障的现代乡村社会治理体制，健全自治、法治、德治相结合的乡村治理体系，增强农村基层组织凝聚力。推动乡村振兴战略与脱贫攻坚、全域旅游、生态文明建设有机结合，实现农民富、农业强、农村美的目标，让乡村成为人人向往的美丽家园。

　　（六）全面深化改革开放，激活发展动力活力。今年是我国改革开放40周年，改革的力度将更大，开放的领域将更广，必须把改革开放作为推动发展的强大动力，着力打造要素洼地、创业福地、开放高地。突出改革重点领域和关键环节。深化供给侧结构性改革，推动要素市场化配置改革，重点在“破、立、降”上下功夫，提高全要素生产率。纵深推进“放管服”改革，最大限度减少审批，多措并举加强监管，不断创新优化服务。抓好商事制度改革，推进工商登记便利化，推行“双随机、一公开”，加强事中事后监管。深化“互联网+政务服务”改革，推行投资建设项目并联审批，完成“两集中两到位”改革，基本形成“一网一线一库两厅两平台”政务服务体系。统筹推进核心景区管理体制改革、国有企业混合所有制改革及盐业、财税、金融、科技、文化、司法、生态、安全生产、城市管理等领域改革，不断释放发展潜力。打造创新创业新引擎。加快发展创业孵化服务，推广创客空间、创新工场等新型孵化模式。推进“互联网+”创业服务，引导、鼓励发展市场化网商创业平台，建设一批小微企业创业创新基地。支持大学生、返乡人员因地制宜创业。开工建设软件大厦，发展第三方专业服务，降低创业门槛和成本，力争新发展网络市场主体同比增长10%。促进开放型经济快速成长。大力发展跨境电商，建设国际公共保税仓、保税商品展示中心、跨境直购中心，实现丰泽保税仓建成营业。加强进境指定口岸、出口食品农产品质量安全示范区建设，发挥国家级生态原产地产品保护示范市品牌优势，实现养殖大鲵出口。继续实施对外贸易“破零倍增”，力争进出口总额增长10%。推进口岸发展，提升通关便利化水平，打造湘西北对外开放门户。

　　（七）提高保障和改善民生水平，加强和创新社会治理。从事关群众切身利益的突出问题入手，重点办好30件民生实事，不断满足人民日益增长的美好生活需要。优先发展教育事业。抓紧抓实县域内城乡义务教育一体化改革发展“十项计划”，新建丽景小学，扩建永定中学、崇实北校、武陵源一中、慈利双岗小学，继续推进“全面改薄”。加快发展学前教育，扩大公办和普惠性民办幼儿园覆盖率。鼓励兴办职业教育、民办教育，积极发展特殊教育和老年教育。启动张家界独立本科院校建设。加快发展医疗卫生事业。深化医药卫生体制改革，建立健全人人共享的基本医疗卫生服务体系。建成市人民医院新院，加快推进18个乡镇中心卫生院、14个社区卫生服务中心、200个医疗点建设。稳妥实施全面两孩政策，促进人口长期均衡发展。加快“健康张家界”建设，开展爱国卫生运动，落实食品安全“四个最严”，加快创建国家食品安全示范城市，全方位全周期保障人民健康。推动文化体育广播电视事业繁荣发展。推进“一宫四中心”、“五馆一院”建设，建成贺龙体育中心。抓好传统村落和少数民族特色村寨保护。加强乡镇综合文化站和村级综合文化服务中心建设，建设市县乡村四级广播平台，进一步提升广播电视综合覆盖率。大力推进文艺创作，努力创作与时代相符的精品力作。扎实开展全民健身，实施文化惠民、体育惠民行动，不断丰富群众文化体育生活。织密扎牢社会保障网。落实积极就业政策，开展职业技能培训2万人次，新增城镇就业1万人、农村劳动力转移就业1.3万人，确保城镇“零就业”家庭动态清零。推进“五大保险”扩面征缴工作，强化低保五保动态管理。健全留守儿童、留守妇女、留守老人和残疾人关爱服务体系，启动建设市级养老服务中心，统筹城乡社会救助体系建设，全面推进“救急难”工作。打造共建共治共享的社会治理格局。推进“三调联动”，预防和化解矛盾纠纷，着力解决历史遗留问题。弘扬优秀传统文化，加强市民文明素质教育，培育自尊自信、理性平和、积极向上的社会心态。加强社区治理体系建设，发挥社会组织作用，实现政府治理和社会调节、居民自治良性互动。健全公共安全应急体制，加强重大决策社会稳定风险评估，有效防范各类突发事件。深化“安全生产监管执法年”活动，强力推进隐患排查治理和“打非治违”，争取不发生较大以上安全事故，确保不发生重特大安全事故。加快社会治安防控体系建设，深化“雪亮工程”管理应用，依法打击和惩治违法犯罪活动。

　　（八）持续改善生态环境，建设“美丽张家界”。良好的生态环境是最普惠的民生福祉，是张家界发展的生命线。我们要像对待生命一样对待生态环境，永葆一方蓝天青山碧水。加大生态系统保护力度。启动生态保护红线划定勘界定标。加强世界自然遗产保护，推进武陵源核心景区移民搬迁。加强大鲵国家级自然保护区建设管理，促进大鲵资源保护与地方经济发展协调统一。落实环境保护“三同时”制度，确保环保设施正常运行，稳定发挥减排效益。全面加强森林防火和林业有害生物防治工作，实施天然林保护工程，完成人工造林8万亩、石漠化治理25.72平方公里。着力解决突出环境问题。抓好中央环保督察后续整改，完成涉自然保护区水电站整改任务。继续开展规范农民建房、集镇环境整治、旅游干线环境整治、环境整治“6+1”四个专项行动，打好大气、水、土壤污染防治“三大战役”。全面推行河长制、路长制，重点加强澧水流域生态环境治理，整治破坏河道、非法采砂、非法取水、超标排污、电毒炸鱼、倾倒垃圾等突出问题；重点加强公路、景区沿线和城乡结合部环境综合治理。采取过硬措施，完成黄标车淘汰任务。加快中心城市、县城、园区污水处理设施建设和改造，推进乡镇环境整治污水处理设施、垃圾处理设施、停车场和农贸市场“四项标配”建设，加强土壤重金属污染治理，力争行政村人居环境整治覆盖率达80%以上。推进绿色发展。坚持节约优先、保护优先、自然恢复为主的方针，形成节约资源和保护环境的空间格局、产业结构、生产方式、生活方式。推进资源全面节约和循环利用，降低能耗、物耗，实现生产系统和生活系统循环链接。加强节能减排，注重能源消耗总量和强度“双控”管理，支持企业利用新技术、新标准开展清洁生产。开展创建节约型机关、环境友好企业、绿色家庭、绿色学校、绿色社区、绿色酒店和绿色出行等行动。

　　（九）加强自身建设，全面提高政府治理现代化水平。开展“不忘初心、牢记使命”主题教育，把全面从严治党要求落实到从严治政之中，努力提升行政能力和服务水平。强化开明开放，打造创新政府。解放思想，更新观念，敢于冲破传统思维、习惯做法，敢于革除影响发展的体制机制，增强开放意识、合作意识和机遇意识，以新时代的新思想、新蓝图、新部署、新要求武装头脑、引领方向、指导实践。以战略思维、创新思维、辩证思维、法治思维、底线思维谋划工作，确保决策科学，经得起实践、历史、人民的检验。改进方法，破解难题，坚持目标导向、问题导向，注重精准精细，静下心研究问题，沉下去解决难题，推动工作落细落小落实。强化依法行政，打造法治政府。严格执行“三重一大”决策程序，健全公众参与、专家论证、风险评估、合法性审查、集体讨论决定制度，推进督查和审计结果公开、决策执行情况公开，确保权力公开透明运行。自觉接受人大及其常委会法律和工作监督、政协民主监督，切实做好建议提案办理工作，主动接受群众监督和媒体监督。加强全民普法工作，提升全民法律素质。以推进政务诚信、商务诚信、社会诚信为重点，加强社会信用体系建设，加快构建“亲”“清”新型政商关系，努力形成法治化便利化的营商环境。防范化解财政金融风险，降低综合债务率。强化履职担当，打造实干政府。办好张家界的事情，关键在人，重在落实。必须按照增强“学习本领、政治领导本领、改革创新本领、科学发展本领、依法执政本领、群众工作本领、狠抓落实本领、驾驭风险本领”的要求，狠抓学习，强化本领，不断提升履职能力。必须强化执行意识和效率意识，坚决整治“门好进、脸好看、事难办”的问题，严格绩效考核，提高行政效能。必须坚持“三个区分”，加强正向激励，为改革者撑腰、为负责者负责、为担当者担当。强化清正严明，打造廉洁政府。坚持把纪律和规矩挺在前面，严格执行《准则》和《条例》，认真贯彻中央八项规定实施细则精神，坚决防止和纠正“四风”问题。持续推进反腐倡廉，从严落实党风廉政建设主体责任和“一岗双责”，聚焦侵犯群众切身利益的“微腐败”，盯紧项目建设、征地拆迁、脱贫攻坚等重点领域，做到有腐必惩、有贪必肃，以政府的“清廉指数”提升人民的“满意指数”。

　　各位代表、同志们，新时代赋予新使命，新起点当有新作为。今年是张家界建市30周年，30年沧桑巨变，30年风雨同舟，让我们更加紧密地团结在以习近平同志为核心的党中央周围，在市委的坚强领导下，不忘初心、牢记使命，开拓奋进、砥砺前行，奋力谱写新时代张家界发展的新篇章，以优异成绩迎接建市30周年！
